# Bilateral dorsolateral prefrontal cortex high-frequency transcranial magnetic stimulation for consciousness recovery after traumatic brain injury: a case series

**DOI:** 10.3389/fpsyt.2025.1642846

**Published:** 2025-10-03

**Authors:** Chun-Hung Chang, Hao-Yu Chuang, Albert Leung, Wei-Sheng Juan, Jia-Hau Lee, Hsin-Chi Tsai

**Affiliations:** ^1^ An-Nan Hospital, China Medical University, Tainan, Taiwan; ^2^ Institute of Clinical Medical Science, China Medical University, Taichung, Taiwan; ^3^ Shu-Zen Junior College of Medicine and Management, Tainan, Taiwan; ^4^ School of Medicine, China Medical University, Taichung, Taiwan; ^5^ Department of Biomedical Engineering, China Medical University, Taichung, Taiwan; ^6^ Translational Cell Therapy Center, Tainan Municipal An-Nan Hospital-China Medical University, Tainan, Taiwan; ^7^ Division of Neurosurgery, Tainan Municipal An-Nan Hospital-China Medical University, Tainan, Taiwan; ^8^ Department of Anesthesiology, School of Medicine, Center for Pain Medicine, University of California, La Jolla, San Diego, CA, United States; ^9^ Center for Pain and Headache Research, VA San Diego Healthcare System, San Diego, CA, United States; ^10^ Department of Psychiatry, Tzu-Chi General Hospital, Hualien, Taiwan; ^11^ Institute of Medical Sciences, Tzu-Chi University, Hualien, Taiwan

**Keywords:** transcranial magnetic stimulation, traumatic brain injury, consciousness recovery, DLPFC (dorsolateral prefrontal cortex), bilateral

## Abstract

**Background:**

Repetitive transcranial magnetic stimulation (rTMS) is a nonpharmacological intervention that can facilitate consciousness recovery after acquired brain injury. However, the effectiveness of high-frequency rTMS applied bilaterally to the dorsolateral prefrontal cortex (DLPFC) and the utility of single-photon emission computed tomography (SPECT) for monitoring treatment response remain unclear.

**Methods:**

Two patients with severe brain injury—one with primary traumatic brain injury and the other with secondary brain injury involving hypoxemic encephalopathy following trauma—received 10Hz rTMS targeting the bilateral DLPFC (Beam-F3/F4). Each session included 40 trains of 4 seconds with 11-second intertrain intervals, delivered at 100% of the resting motor threshold. Sessions were administered daily, 5 days a week, with 10 sessions per course.

**Results:**

Both patients exhibited clinical improvement, with Glasgow Coma Scale scores increasing from 6 to 10 and Coma Recovery Scale–Revised scores increasing from 6 to 16 after 12 courses, indicating a transition from a vegetative state to a minimally conscious state. SPECT revealed reduced hypoperfusion in the bilateral frontal lobes, decreasing from 51% to 40% in Patient A and from 33% to 30% in Patient B. These imaging findings are consistent with the observed clinical improvements.

**Conclusion:**

High-frequency rTMS applied bilaterally to the DLPFC may promote consciousness recovery in patients with acquired brain injury, with associated perfusion improvements observed on SPECT. Although these findings are promising, additional controlled studies in larger cohorts are required for validation.

## 1 Introduction

Each year, approximately 50 to 60 million new cases of traumatic brain injury (TBI) occur worldwide, and approximately 3.5 million of these cases are reported in the United States alone (1). TBI is among the most common causes of various neurological conditions (1) and often results in disorders of consciousness (2). These disorders are characterized by altered states of consciousness and are subdivided into coma, vegetative state, and minimally conscious state (3). Pharmacological treatments, such as amantadine, are used to treat these disorders; however, the effectiveness of such treatments remains limited (4). Many nonpharmacological interventions have been developed to facilitate consciousness recovery (3). Among major nonpharmacological neuromodulatory interventions, repetitive transcranial magnetic stimulation (rTMS) is regarded as a versatile option because of its noninvasiveness and precision in cortical stimulation (5).

The nonpharmacological intervention rTMS noninvasively stimulates the brain by using electromagnetic coils to generate small focal electrical currents in the cortex (6, 7). The frequency of rTMS can be classified as high or low. Low-frequency stimulation (<5Hz) exerts an inhibitory effect and promotes long-term depression–like plasticity, whereas high-frequency stimulation (≥5 Hz) induces cortical excitation and long-term potentiation–like plasticity (8). Pilot studies have highlighted the potential of rTMS for consciousness recovery. A meta-analysis (5) of five randomized controlled trials [7–11] evaluated the efficacy of rTMS in promoting consciousness recovery. The meta-analysis reported a small but significant effect size (mean difference: 1.59; 95% confidence interval [CI]: 1.01–2.18; *p*<0.01). Another meta-analysis (Yang et al. (9) of seven randomized controlled trials (10–16) revealed that rTMS significantly promoted recovery compared with the findings in the control group, with a weighted mean difference (WMD) of 1.89 in Coma Recovery Scale–Revised (CRS-R) scores (95% CI: 1.39–2.39; *p*<0.00001). Subgroup analysis revealed that rTMS targeting the dorsolateral prefrontal cortex (DLPFC) led to a greater improvement in CRS-R scores (WMD: 2.24; 95% CI: 1.55–2.92; *p*<0.00001; *I* ^2^ = 31%) than did rTMS targeting the primary motor cortex (WMD: 1.63; 95% CI: 0.69–2.57; *p*=0.0007; *I* ^2^=14%). Although studies have demonstrated the efficacy of rTMS applied to the unilateral DLPFC for consciousness recovery (10, 14, 17), the efficacy of high-frequency rTMS applied bilaterally to the DLPFC for post-TBI consciousness recovery remains unclear. Furthermore, the utility of brain perfusion single-photon emission computed tomography (SPECT) in evaluating responses to rTMS and its potential as a biomarker of post-TBI consciousness recovery remains to be established. The present article describes two cases where high-frequency rTMS was applied bilaterally to the DLPFC for post-TBI consciousness recovery, and SPECT evaluations were conducted both before and after the rTMS treatment.

## 2 Case presentation

### 2.1 Patient A

**Figure 1:**
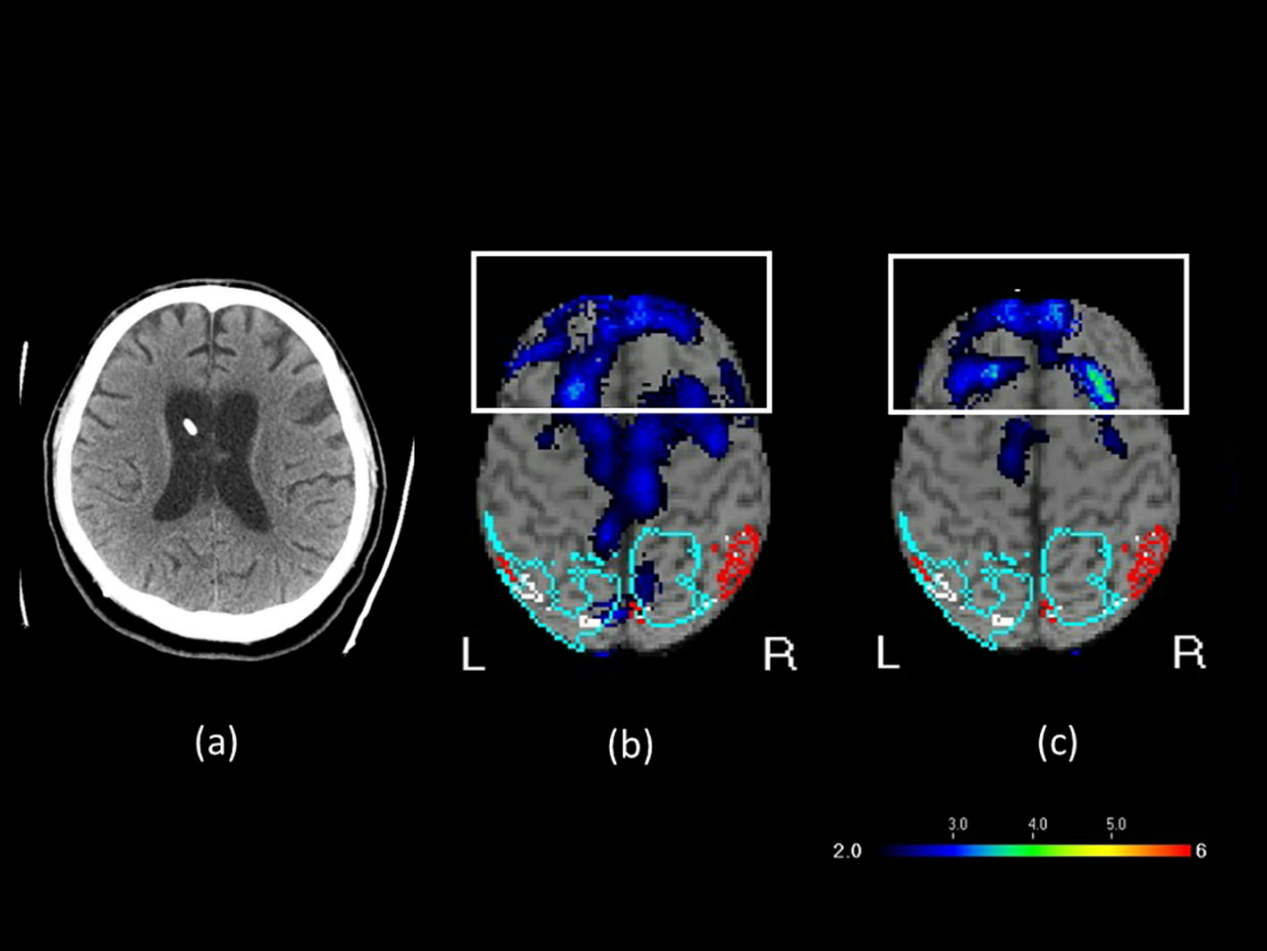
**(a)** Brain CT image before rTMS treatment, **(b)** SPECT brain image before rTMS treatment, and **(c)** SPECT brain image after rTMS treatment in Patient A. In the transverse SPECT view, the white rectangular box highlights the frontal lobe. The color scale represents varying levels of blood flow or functional activity, with cooler colors such as blue and purple indicating regions of hypoperfusion.

A 42-year-old man was injured in a traffic accident in May 2021. The injury resulted in a traumatic subdural hematoma, subarachnoid hemorrhage, and intracerebral hemorrhage. He was left in a vegetative state with a Glasgow Coma Scale (GCS) score of 6, consisting of eye response (E1; no eye opening), motor response (M4; withdrawal response to pain), and verbal response (V1; no verbal response) (18). His Coma Recovery Scale–Revised (CRS-R) (19) score was 6. CRS-R scores were retrospectively derived from detailed clinical charts and behavioral records documented during hospitalization. Two senior physicians independently reviewed the records after data collection and assigned scores based on the documented behaviors, without blinding to outcomes. He received multiple therapies, such as rehabilitation, traditional Chinese acupuncture, and hyperbaric oxygen therapy, but exhibited no substantial improvements in consciousness. In April 2022, he was referred to our center for transcranial magnetic stimulation (TMS) evaluation. The patient exhibited sleep–wake cycles without awareness. Motor responses were characterized by purposeless movements, posturing, or withdrawal in response to noxious stimuli. Both auditory and visual responses were limited to startle reactions. No evidence of communication was noted, and emotional responses were reflexive. He occasionally groaned in response to pain, particularly at night. A brain computed tomography (CT) scan revealed bilateral diffuse white matter hyperintensities, consistent with axonal injury due to TBI, and the presence of a right-sided ventricular drainage catheter used for intracranial pressure management. A shunt valve adjustment tool (Medtronic, USA) displayed no changes after rTMS treatment (**Supplementary Figure S1**). Brain perfusion was assessed through SPECT (GE Discovery NM/CT 670) with Tc-99m ethyl cysteinate dimer (ECD). Images were analyzed using the Easy Z-score imaging system, which compares patient scans with those from an age-matched normal database. In the transverse SPECT view, cooler colors such as blue and purple indicated regions of hypoperfusion, representing areas of reduced blood flow or attenuated functional activity in the brain. Tc-99m ECD brain perfusion SPECT revealed reduced activity in the bilateral inferior and superior frontal lobes, cingulate, precuneus, and left occipital areas (**Figure 1**). Patient A was maintained on piracetam 2400 mg/day for cognitive support, acetaminophen 2000 mg/day for pain control, bisacodyl 10 mg/day for constipation, bisoprolol 2.5 mg/day for tachycardia, and tizanidine 6 mg/day for spasticity. These medications and their dosages had remained unchanged for more than 3 months before the initiation of rTMS and throughout the treatment period. He had no contraindications to rTMS and no history of seizure before or during hospitalization.

After informed consent was obtained from the patient’s family, the patient was subjected to multiple rTMS sessions. (9) suggested that high-frequency rTMS targeting the DLPFC is more effective than that targeting the primary motor cortex in improving consciousness. Therefore, we administered high-frequency rTMS bilaterally to the DLPFC. Coil positioning was determined using the algorithm developed by Beam et al. (20), with the Beam-F3 position used for the left DLPFC and the Beam-F4 position for the right DLPFC (20, 21). An Apollo TMS therapy stimulator (MAG & More, Germany) equipped with a figure-of-eight coil was used. On the basis of the literature (5, 22) and established safety standards, we adopted a modified protocol consisting of 40 trains at 10Hz, with each train comprising 4 seconds of stimulation followed by 11 seconds of rest, for a total duration of 10 minutes at 100% of the resting motor threshold. The threshold was determined by stimulating the primary motor cortex and identifying the minimum intensity that elicited a motor-evoked potential (23, 24). Stimulation was applied first to the left DLPFC and then to the right DLPFC (3,200 pulses per session). This treatment sequence was determined on the basis of the findings of relevant studies on TBI-related depression (25, 26). The coil was oriented at 45° to the sagittal midline to optimize stimulation of the DLPFC (27, 28). Treatment was delivered once a day, 5 days a week, with 10 sessions per course.

During the eighth course of treatment, a substantial improvement was observed in the range of motion of the left hand. The left hand, which had initially hung by the abdomen, could be raised to chest level. During the ninth course, hand and foot movements improved further during rehabilitation sessions. By the twelfth course, the patient responded with laughter when the family played a humorous television program, “Iron Lion Jade Dragon.” In addition, the patient became more responsive to sounds, turning toward speaking family members, and opened his eyes and smiled at jokes. Repeat clinical evaluation indicated that the patient’s level of consciousness had improved from a vegetative state to a minimally conscious state, with a GCS score of 10 (E4M4V2) and a CRS-R score of 16 (**Table 1**). The patient also exhibited sleep–wake cycles with partial awareness, purposeful motor responses localizing to noxious stimuli, auditory localization to sound, sustained visual fixation or pursuit, intelligible verbal or gestural communication, and contingent emotional responses. To evaluate treatment-induced changes, brain perfusion. SPECT was performed within 1 week from the twelfth course of rTMS. SPECT (**Figure 1b**) revealed 51% reduction in perfusion in the bilateral inferior and superior frontal lobes, cingulate, precuneus, and left occipital areas. This finding aligned with electroencephalography (EEG) results suggesting mild diffuse cortical dysfunction after the TBI. After rTMS, the degree of hypoperfusion decreased to 40% (**Figure 1c**). Patient A subsequently received four additional rTMS courses; however, no further substantial gains were observed, indicating a plateau. The patient’s family therefore opted for a maintenance protocol, with treatment administered two to three times per week, given the financial burden of continual out-of-pocket expenses.

**Table 1:** Clinical and neuroimaging outcomes after 12 sessions of rTMS in patients with TBI-related disorders of consciousness.

Case	Age	Sex	GCS before rTMS	GCS after 12-course rTMS	CRS-R before rTMS	CRS-R after 12-course rTMS	SPECT before rTMS (hypoperfusion area in the bilateral frontal lobe)	SPECT after 12-course rTMS (hypoperfusion area in the bilateral frontal lobe)
A	42	Male	6	10	6	16	51%	40%
B	30	Male	6	10	6	16	33%	30%

### 2.2 Patient B

**Figure 2:**
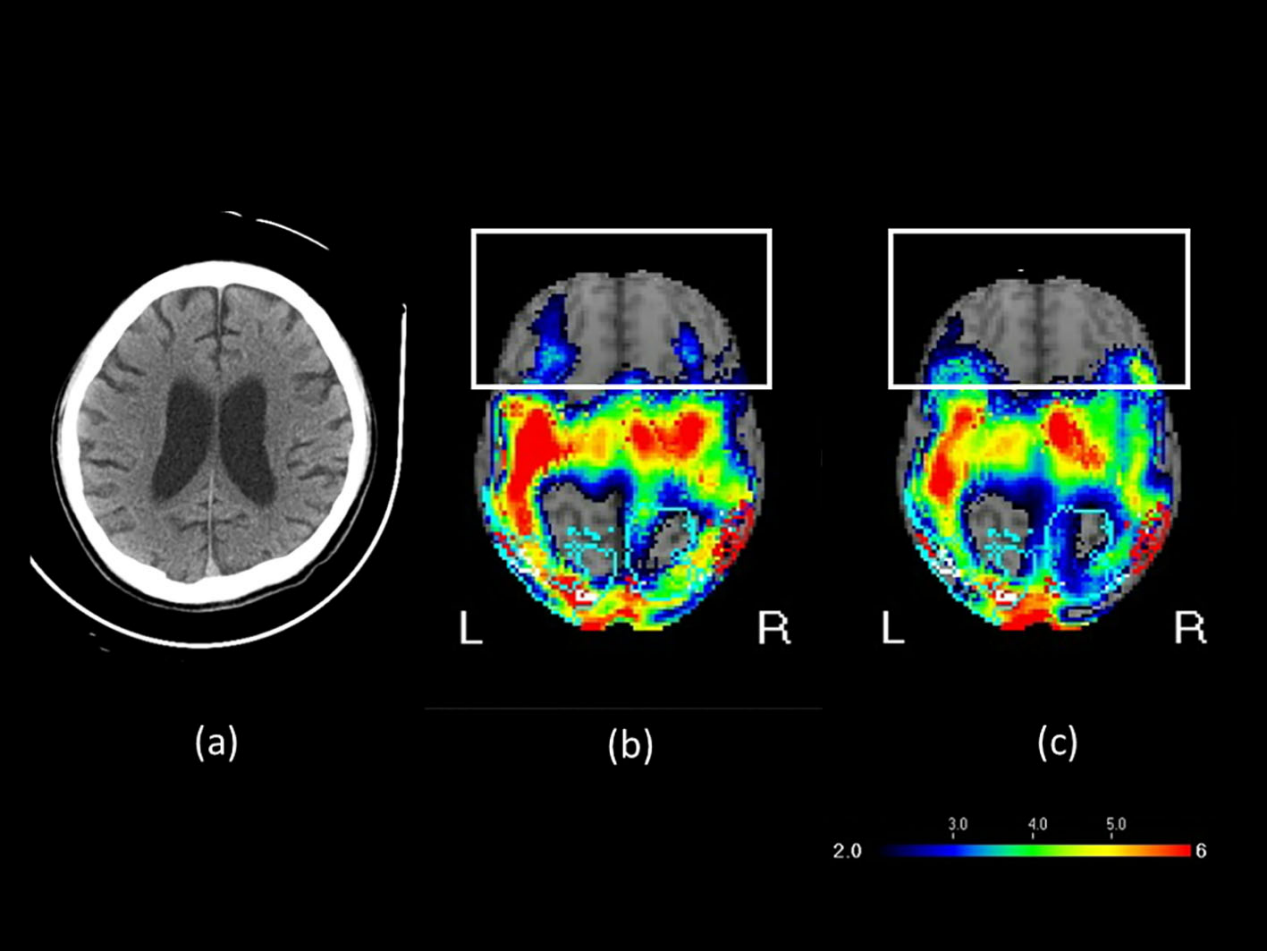
**(a)** Brain CT image before rTMS treatment, **(b)** SPECT brain image before rTMS treatment, and **(c)** SPECT brain image after rTMS treatment in Patient B. In the transverse SPECT view, the white rectangular box highlights the frontal lobe. The color scale indicates varying levels of blood flow or functional activity, with cooler colors such as blue and purple representing regions of hypoperfusion.

A 30-year-old male sustained a severe brain injury following a motor vehicle accident on May 22, 2021, which resulted in multiple rib fractures, hemothorax, liver laceration, and splenic rupture. The massive hemorrhage led to circulatory collapse and an out-of-hospital cardiac arrest (OHCA), for which cardiopulmonary resuscitation (CPR) was performed. The subsequent hypoxemic encephalopathy was therefore considered a consequence of secondary brain injury, as the ischemic and hypoxic damage was directly attributable to the primary traumatic insult. Secondary brain injury is a well-recognized complication of severe trauma and includes ischemic and hypoxic damage, cerebral edema, elevated intracranial pressure, hydrocephalus, and infection (29, 30). He underwent various therapies, such as rehabilitation, traditional Chinese acupuncture, and hyperbaric oxygen therapy, but remained in a vegetative state. In March 2022, after 10 months in this state, he was referred to our brain stimulation outpatient clinic for rTMS evaluation. The patient’s GCS was 6 (E2V1M3) and CRS-R was 6, indicating severe impairment of consciousness. The patient exhibited sleep–wake cycles without awareness. Motor responses were characterized by purposeless movements, posturing, or withdrawal in response to noxious stimuli. Both auditory and visual responses were limited to startle reactions. He occasionally groaned or became agitated, twisting his body unconsciously. A brain CT scan revealed communicating hydrocephalus and mild diffuse brain atrophy. Tc-99m ECD brain perfusion SPECT revealed reduced activity in the bilateral parietal, precuneus, occipital, superior frontal, thalamic, posterior cingulate, left inferior frontal, and inferior-anterior temporal areas (**Figure 2**). Patient B was maintained on amantadine 200 mg/day and piracetam 2400 mg/day for cognitive support, acetylcysteine 1200 mg/day as a mucolytic for sputum clearance, propranolol 45 mg/day for tachycardia, flunarizine 10 mg/day for improved peripheral vascular circulation, and baclofen 30 mg/day for spasticity. These medications and their dosages remained unchanged for more than 3 months and throughout the treatment period. He had no contraindications to rTMS and no history of seizures.

After informed consent was obtained from the patient’s family, the patient was subjected to multiple sessions of high-frequency rTMS applied bilaterally to the DLPFC. The treatment protocol was the same as that used for Patient A.

During the seventh course of treatment, the patient exhibited signs of anger. By the tenth course, he exhibited crying and tearful behavior. During the twelfth course, the patient gradually began to respond to calls from family members by turning his head toward the sound. He would actively open his eyes when called and smiled when family members joked with him. During treatment, the patient followed commands from family members to keep his head still during TMS delivery. The patient’s level of consciousness improved from a vegetative state to a minimally conscious state, with the GCS score increasing from 6 to 10 (E4M4V2) and the CRS-R score improving from 6 to 16 (**Table 1**). He also exhibited sleep–wake cycles with partial awareness, purposeful motor responses localizing to noxious stimuli, auditory localization to sound, sustained visual fixation or pursuit, intelligible verbal or gestural communication, and contingent emotional responses. **Figure 2** presents the brain imaging results of Patient B before and after rTMS treatment. The level of hypoperfusion detected by SPECT decreased from 33% to 30%. EEG revealed diffuse motion artifacts in both hemispheres due to patient discomfort. Patient B underwent six additional rTMS courses; however, no further substantial clinical improvement was observed. He was subsequently transitioned to home-based care. Because of the out-of-pocket expenses associated with transportation and treatment, the family opted for a maintenance therapy schedule, with sessions administered two to three times a week.

## 3 Discussion

### 3.1 Principal findings versus the literature

To the best of our knowledge, this is the first study to report that high-frequency rTMS applied bilaterally to the DLPFC can promote post-TBI consciousness recovery. Severe brain injuries can disrupt neural networks responsible for maintaining arousal and awareness, which are key components of consciousness. In patients with severe brain injuries, abnormal connectivity is observed in the default mode network (DMN) and other cortical and subcortical networks essential for consciousness. The extent of altered connectivity is associated with the severity of consciousness impairment, and the recovery of consciousness is associated with the restoration of these neural connections (31). Disruption of the DMN may cause disorders of consciousness (32). The DLPFC is a central hub for executive functions and plays a vital role in regulating networks involved in cognitive control, attention, and consciousness (33–35). Targeting the DLPFC with rTMS can help regulate connectivity in the DMN (3, 13, 36). A study demonstrated that 10-Hz excitatory TMS over the left DLPFC normalized depression-related subgenual hyperconnectivity in the DMN (37). The DLPFC, as a key component of the executive control network (ECN), also mediates environmental awareness and restores the ECN–DMN balance (38). Therefore, stimulation of the DLPFC may modulate internetwork connectivity between the ECN and DMN through the salience network, facilitating transition from a vegetative state or unresponsive wakefulness syndrome to a minimally conscious state.

In addition to stimulation of the left DLPFC, rTMS targeting the right DLPFC has been investigated for consciousness recovery. A study reported that rTMS applied to the right DLPFC mitigated consciousness disturbances in 10 patients with stroke, as evidenced by the results of quantitative EEG spectral power analysis (39). Another study involving 10 healthy individuals and 10 patients with postanoxic unresponsive wakefulness syndrome indicated that a single session of 10-Hz rTMS applied to the right DLPFC transiently improved consciousness and partially restored connectivity in several cortical areas in some patients with unresponsive wakefulness syndrome (40). Furthermore, in a study examining 32 patients in a vegetative state due to brain injury, 10-Hz rTMS applied to the right DLPFC for 20 days significantly increased CRS-R scores compared with pretreatment scores (p < 0.001) (12). On the basis of these findings, we combined stimulation of the left DLPFC with that of the right DLPFC. We hypothesized that bilateral DLPFC stimulation would be more effective than unilateral DLPFC stimulation in promoting consciousness recovery. This case series provides early evidence supporting this hypothesis.

The 10-Hz stimulation frequency was selected on the basis of both empirical evidence and clinical experience. A meta-analysis of three studies (9, 10, 12, 15) highlighted the potential of 10-Hz rTMS in treating disorders of consciousness. Our clinical team has extensive experience in using 10-Hz protocols for treating major depressive disorder; this experience supports the safety and feasibility of 10-Hz rTMS. The stimulation duration —10 minutes per hemisphere targeting the left and right DLPFC—was selected to align with the approximately 20-minute total session length reported in relevant studies (10, 12, 15). The bilateral approach ensures balanced modulation of both hemispheres and is associated with improved outcomes in consciousness recovery. The stimulation intensity was set to 100% of the resting motor threshold, consistent with parameters commonly adopted in studies involving patients with impaired consciousness (17, 41). This intensity is considered sufficient to achieve cortical activation while maintaining safety.

In our cases, SPECT revealed reduced hypofunction in the prefrontal cortex after bilateral high-frequency rTMS applied to the DLPFC. Therefore, this approach can enhance prefrontal cortical perfusion in patients with severe brain injury. SPECT has previously been used to evaluate TBI (42). The posttreatment reduction in hypofunction in the prefrontal cortex may indirectly reflect improved neural activity and connectivity. This observation aligns with the literature highlighting the role of the DLPFC in cognitive recovery and consciousness (2, 5, 9). The observed improvement in brain perfusion supports the potential of bilateral high-frequency rTMS targeting the DLPFC to effectively enhance brain function and support recovery in patients with impaired consciousness. Additional studies are required to confirm these results and elucidate the underlying mechanisms.

### 3.2 Safety and adverse effects

No seizure was observed in our cases. The reported risk of seizure in patients with severe TBI (GCS < 9) is 0.5% (54,545 patients) (43). A meta-analysis indicated that rTMS is associated with an elevated risk of minor adverse effects, such as headache, pain, dizziness, drowsiness, and dry mouth; however, these symptoms typically resolve soon after discontinuation of rTMS (9). Overall, rTMS appears to be a safe and well-tolerated intervention suitable for clinical application.

### 3.3 Limitations

This case report has several limitations. Only two patients were evaluated. Larger studies with randomized designs are required to verify the effects of 10-Hz rTMS applied bilaterally to the DLPFC. Furthermore, EEG assessments were not performed before rTMS. EEG provides real-time information on cortical excitability, brainwave patterns, and neuronal activity, whereas SPECT provides data on brain perfusion. However, SPECT offers superior spatial resolution than does EEG, enabling the detailed imaging of deep brain structures and detection of abnormalities in cerebral blood flow. These advantages make SPECT particularly useful for evaluating functional brain activity, especially in disorders involving complex brain regions, whereas the use of EEG remains limited to examining electrical activity on the brain’s surface. Future investigations should incorporate both assessments for a comprehensive evaluation. Another limitation of this report is that the CRS-R scores were obtained retrospectively from clinical documentation rather than through prospective standardized assessments. Although the scoring was conducted by two senior physicians, the retrospective nature and lack of blinding may reduce the precision of the assessments. Future studies should incorporate prospective, standardized CRS-R evaluations to enhance reliability and comparability across reports.

## 4 Conclusion

Our findings suggest that high-frequency rTMS applied bilaterally to the DLPFC may improve consciousness recovery in patients with TBI. SPECT revealed improved brain perfusion after rTMS treatment. Nonetheless, our findings should be validated in future studies with larger sample sizes, randomized double-blind designs, and placebo-controlled protocols.

## Data Availability

The original contributions presented in the study are included in the article/**Supplementary Material**. Further inquiries can be directed to the corresponding author.

## Supplementary material

Supplementary Figure 1 Shunt valve adjustment tool exhibiting no changes after rTMS treatment.
