# Supplementary figures and images for: Bilateral dorsolateral prefrontal cortex high-frequency transcranial magnetic stimulation for consciousness recovery after traumatic brain injury: a case series
Source: Front Psychiatry. 2025 Oct 3;16:1642846. doi: 10.3389/fpsyt.2025.1642846 (PMC12532002; doi:10.3389/fpsyt.2025.1642846)

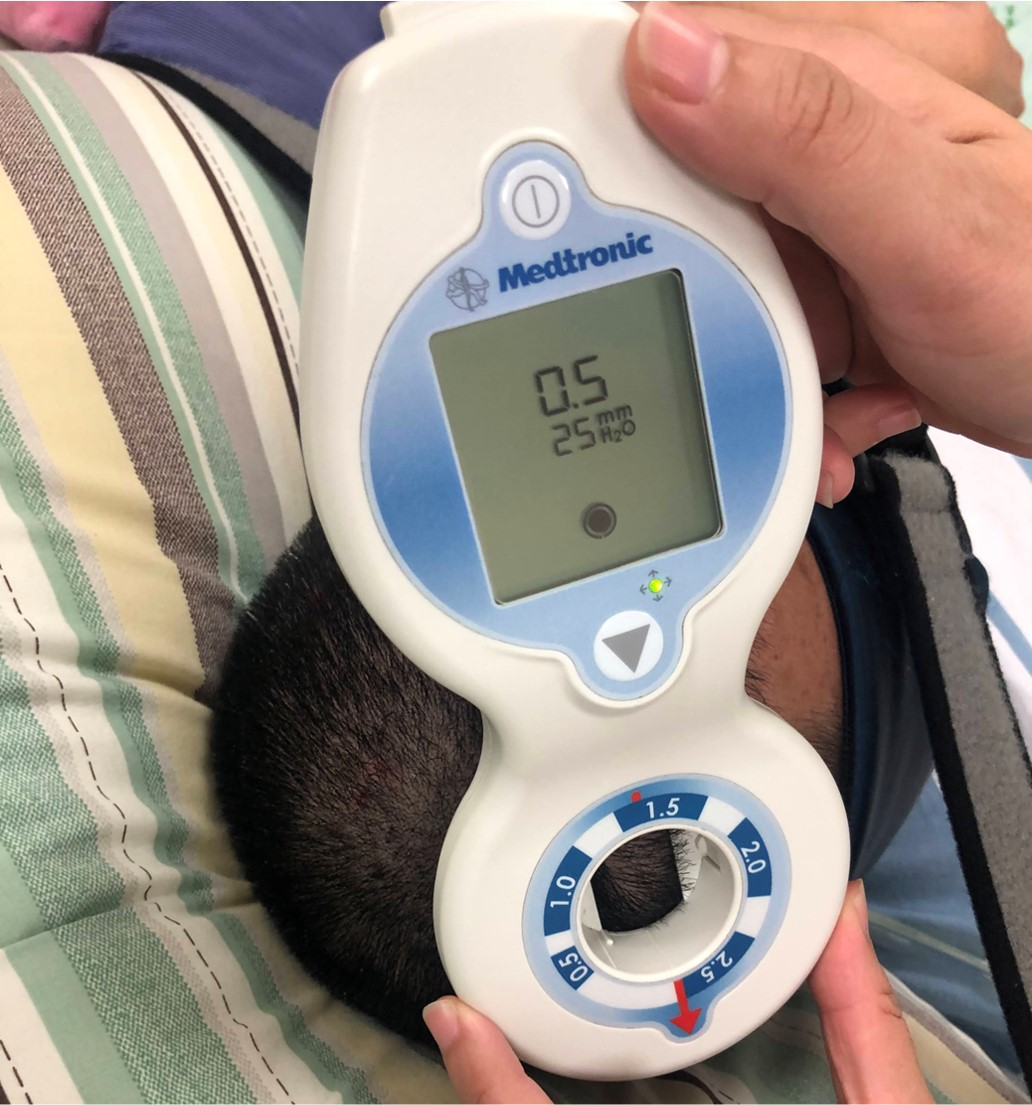

Supplement: Supplementary Figure 1 — Shunt valve adjustment tool exhibiting no changes after rTMS treatment. [file Image1.tif]
